# Breeding for resistance to gastrointestinal nematodes – the potential in low-input/output small ruminant production systems

**DOI:** 10.1016/j.vetpar.2016.05.015

**Published:** 2016-07-30

**Authors:** P.I. Zvinorova, T.E. Halimani, F.C. Muchadeyi, O. Matika, V. Riggio, K. Dzama

**Affiliations:** a Department of Animal Sciences, University of Stellenbosch, Private Bag X1, Matieland, 7602, South Africa; b Department of Para-clinical Veterinary Studies, University of Zimbabwe, P.O. MP167, Mt. Pleasant, Harare, Zimbabwe; c Department of Animal Science, University of Zimbabwe, P.O. MP167, Mt. Pleasant, Harare, Zimbabwe; d Biotechnology Platform, Agriculture Research Council Private Bag X5, Onderstepoort, 0110, South Africa; e The Roslin Institute and R(D)SVS, University of Edinburgh, Easter Bush, MidlothianEH25 9RG, UK

**Keywords:** Nematode resistance, Selection, Faecal egg counts, SNPs, Small ruminants

## Abstract

The control of gastrointestinal nematodes (GIN) is mainly based on the use of drugs, grazing management, use of copper oxide wire particles and bioactive forages. Resistance to anthelmintic drugs in small ruminants is documented worldwide. Host genetic resistance to parasites, has been increasingly used as a complementary control strategy, along with the conventional intervention methods mentioned above. Genetic diversity in resistance to GIN has been well studied in experimental and commercial flocks in temperate climates and more developed economies. However, there are very few report outputs from the more extensive low-input/output smallholder systems in developing and emerging countries. Furthermore, results on quantitative trait loci (QTL) associated with nematode resistance from various studies have not always been consistent, mainly due to the different nematodes studied, different host breeds, ages, climates, natural infections versus artificial challenges, infection level at sampling periods, among others. The increasing use of genetic markers (Single Nucleotide Polymorphisms, SNPs) in GWAS or the use of whole genome sequence data and a plethora of analytic methods offer the potential to identify loci or regions associated nematode resistance. Genomic selection as a genome-wide level method overcomes the need to identify candidate genes. Benefits in genomic selection are now being realised in dairy cattle and sheep under commercial settings in the more advanced countries. However, despite the commercial benefits of using these tools, there are practical problems associated with incorporating the use of marker-assisted selection or genomic selection in low-input/output smallholder farming systems breeding schemes. Unlike anthelmintic resistance, there is no empirical evidence suggesting that nematodes will evolve rapidly in response to resistant hosts. The strategy of nematode control has evolved to a more practical manipulation of host-parasite equilibrium in grazing systems by implementation of various strategies, in which improvement of genetic resistance of small ruminant should be included. Therefore, selection for resistant hosts can be considered as one of the sustainable control strategy, although it will be most effective when used to complement other control strategies such as grazing management and improving efficiency of anthelmintics currently.

## Highlights

• Resistance to anthelmintics in small ruminants is documented worldwide.
• Genetic resistance in GIN have been studied in experimental and commercial flocks.
• Limited in low-input low-output farming systems.
• Nematode eradication has evolved to manipulation of host-parasite equilibrium.
• Selection for resistant hosts can be considered a sustainable control strategy.

## 1 Introduction

Small ruminants make important contributions to human livelihoods in developing economies. In 2012, 37 and 22% of the 1.2 billion world sheep population together with 56 and 30% of the approximately 1 billion world goat population were located in Asia and Africa respectively (FAO, 2015). In most low-input/output smallholder farming systems they serve as household assets with multiple livelihood functions, providing food, income and important non-market services (Ruto et al., 2008). However, gastrointestinal parasitic infestations impose severe constraints on small ruminant production in marginal systems (Periasamy et al., 2014). Control strategies worldwide are based on the use of anthelmintic drugs, which have often been associated with cases of multiple drug resistant parasites and drug residues in the food and environment. However, most small ruminant farmers in the tropics and sub-tropics are resource-constrained, and do not have access to either anthelmintics or land management practices to mitigate the influence of gastrointestinal nematodes (GIN). Therefore, there is a need for alternative methods of parasite control in these farming systems, with genetic improvement offering a more sustainable option. Although resistance to GIN is well studied in both experimental (Davies et al., 2006, Riggio et al., 2013) and commercial flocks (Matika et al., 2011), a few studies have focused on low-input/output smallholder systems in developing countries. This review offers an overview of current practices and potential control methods for GIN resistance.

### 1.1 Non-genetic methods of internal parasite control

Gastrointestinal nematode control methods previously proposed include chemical, management or biological approaches (Jackson and Miller, 2006). Chemical control is the most widely used method. Alternative approaches, such as use of copper oxide wire particles, have been reported in the control of *Haemonchus contortus* in small ruminants (Torres-Acosta and Hoste, 2008). Copper toxicity is however a problem particularly in sheep (Hoste and Torres-Acosta, 2011), but the potential risk is lower in goats. Use of ethno-veterinary products, dietary and nutritional supplementation have also been reported to reduce parasite infections (Hoste et al., 2006, Terrill et al., 2009). Paolini et al. (2003) reported a reduction by 50–60% in faecal egg counts (FEC) in small ruminants following condensed tannin-rich diets supplementation. However, some condensed tannin extracts have been found to reduce small intestine burdens (*Trichostrongylus colubriformis, Cooperia, Nematodirus, Bunostomum spp*) but not those from the abomasum (*H. contortus, Teladorsagia circumcinta*) (Athanasiadou et al., 2001). Anti-parasitic action has been also demonstrated in chicory (*Cichorium intybus*), sulla (*Hedysarum coronarium*), sainfoin (*Onobrychus viciifolia*) and sericea lespedeza (*Lespedeza cuneata*) (Houdijk et al., 2012). Biological control methods using nematophagous microfungus *Duddingtonia flagrans* have the ability to break the lifecycle of parasites by trapping and killing infective GIN larvae in faeces before they migrate to pasture (Terrill et al., 2012). Rotational resting and grazing as a means of parasite control limits the host-parasite contact thus reducing pasture contamination and increasing productivity in common grazing rangelands. The strategy of rotational resting and grazing is considered as being either preventative, evasive or diluting (Jackson and Miller, 2006). According to Cabaret et al. (2002) and Younie et al. (2004), the preventative strategy involves turning out parasite-free animals on clean pastures. The evasive strategy involves moving animals from contaminated to clean pastures within the same season and alternating grazing of different species. The diluting strategy allows worm challenge to be relieved by diluting pasture infectivity by reducing stocking rates, allowing mixed species grazing of animals of different age groups. However, these above mentioned methods are difficult to apply especially in extensive production systems and in systems with common grazing.

Improved nutrition through supplementation of by-pass protein in small ruminants improves resistance and resilience to GIN (Torres-Acosta et al., 2012). Studies by Steel (2004), Colvin et al. (2012) and Marume et al. (2012) provided evidence of the benefits of protein supplementation as a means of parasite control.

Internal parasites can be controlled by use of vaccines. Some of these vaccines are based on antigens of the parasite stage that adheres to the gut wall and these antigens induce immune responses that interfere with successful attachment in the gut. One of the vaccination methods for example, focuses on identifying protective hidden antigens derived from the worm’s intestinal gut cells (Terrill et al., 2012). When the parasites feed on the host they ingest antibodies that bind to functional proteins on the brush border of their intestinal cells, so that the digestive processes are compromised, leading to starvation, loss of fecundity and weakness. Eventually, the parasites detach and are lost from the predilection site (Jackson and Miller, 2006). Until recently, the use of hidden antigens was only thought to be effective on the cestodes (Waller and Thamsborg, 2004) and not on nematodes. In 2014, a new vaccine against *H. contortus* (Barbervax^®^) was commercially available. This is an alternative to the drench–based control method and it has the ability to manage drench resistance (Maxwell, 2015). The problem associated with the use of this vaccine could be related to cost, i.e. for initial use in an animal, three priming doses are required to achieve an effective level of antibody protection and this protection lasts only approximately 6 weeks; thus an animal will need to be vaccinated again, leading to 4–5 vaccinations being annually required. This poses problems in low-input/output farming systems not only in terms of cost but also for vaccine storage (limited refrigeration capacity) and handling.

The main constraint for the use of anthelmintics (AHs) is the development of drug resistance, which may be a consequence of host-pathogen co-evolution, in which the parasites survive exposure to standard recommended doses of AH and are able to thrive and reproduce under existing dosing regimes. The frequency and dosage of treatment are usually the main factors driving development of resistance to AHs. However, under dosing, which is a common practice in under resourced smallholder farms, particularly in goats, may be the one of the leading forces to parasite resistance. The continuous development of new classes of AH has for several decades compensated for parallel development of resistance (von Samson-Himmelstjerna and Blackhall, 2005), in several genera such as *Haemonchus*, *Trichostrongylus* and *Ostertagia* spp (Kaplan, 2004, McKellar and Jackson, 2004) in sheep and goats. Examples drawn worldwide of AH resistance across chemical compound classes in small ruminants are summarised in Table 1.

## 2 Genetic control of GIN

The genetic control methods involve selection of individuals resistant to GIN (Vagenas et al., 2002) and this relies on the existence of host genetic variation and the predominating environmental conditions. Most goat breeds that are highly resistant to parasite infections are found in the tropics reared under extensive farming (Hohenhaus and Outteridge, 1995), but these breeds remain greatly under-utilised (Baker, 1998). A few studies were conducted on breeding for resistance to GINs in the tropics and subtropics. These include work conducted in Kenya by Baker et al. (1998) in goats (Small East African and Galla breeds) and sheep (Red Masaai and Dorper breeds) and also work conducted in Zimbabwe by Matika et al. (2003) in sheep (Sabi and Dorper breeds). To date, little work has been undertaken in utilising these genetic resources as a means of parasite control via selection and breeding for the resistant lines. Although breeding for GIN resistance is an appealing technique, such approaches are difficult to implement in low-input/output smallholder farming systems, mainly due to lack of record keeping and pedigree data in the open mating systems. This aspect will be cover in another section of this review.

### 2.1 Genetic resistance to parasites, from a classical selection approach

Gastrointestinal parasite resistance is under genetic control and the existence of genetic variation among individuals with regards to resistance to GINs has been studied extensively (Table 2). Conventional breeding strategies are based on the use of indicator traits such as FEC and packed cell volumes (PCV), which are costly and time consuming to collect. Whilst FEC have been the main indicator for resistance to GINs, significant levels of infection are required for genetic variation in FEC to be expressed and in drier parts of the world, this increase in FEC may not occur for several years, or may be masked by parasite control measures aimed at limiting the infection. Nematode resistance assessed by using FEC has a low to high heritability in small ruminants, ranging from 0.01 to 0.65 (Table 3). The heritability of a trait indicates the potential of obtaining genetic gain through selection (Lôbo et al., 2009). For example, selecting animals with the lowest FEC would increase host resistance to parasites. However, resilient animals are not targeted by this approach. Hence, selection and breeding for resistance to GINs is feasible; and a case example of 69% reduction in FEC following genetic selection was reported by Eady et al. (2003). Although selection for resistance is possible and effective for sheep and goats; this has not been fully adopted in most developing countries, but restricted to research flocks, due to complexity in collecting phenotypes and pedigree information and limitations associated with costs involved in running the breeding schemes. Moreover, there are other factors to be taken into account. Technical and infrastructural related issues, for example, are the greatest bottlenecks in genetic improvement programmes for most of the sheep and goat farming systems: small flock sizes, lack of clear breeding goals, lack of or poor infrastructures. These are all factors that contribute to the low participation of farmers in breeding schemes, which in turn makes achieving within-breed genetic improvement highly challenging. It has to be kept in mind, however, that the implementation of a breeding programme requires an accurate pedigree. It has been indeed shown that even in dairy cattle, which have well established breeding programme, over 20% of registered animals have paternity errors (Ron et al., 1996) and this percentage is probably even higher in small ruminants. In smallholder properties in tropical and subtropical environments usually there is no pedigree recording and no data recording at any time. Mating systems are often not planned with all year round kidding/lambing with community animals mixing in communal shared grazing lands. This renders the conventional breeding practices as we know them currently impossible to implement. However, there are other possibilities with the modern technologies that may remedy some of these shortfalls.

### 2.2 Identification of QTL associated with GIN resistance

Quantitative trait loci (QTL) mapping can help in understanding the complexity of parasite resistance by identifying candidate genomic regions. Studies using microsatellite markers (Beh et al., 2002, Davies et al., 2006, Gutiérrez-Gil et al., 2009, Marshall et al., 2009) have been conducted to understand the mechanisms underlying parasite resistance. Candidate gene studies, together with microarray and gene association studies have also been conducted in several small ruminant breeds in an effort to identify genes that are involved in the control of resistance and susceptibility (Crawford et al., 2006, Brown et al., 2013). The candidate gene approach focuses on identifying DNA markers within candidate genes, which may not necessarily be causative mutations for resistance themselves, but may be in linkage disequilibrium (LD) with the causative mutation (Sayers and Sweeney, 2005). Candidate genes implicated included those that regulate the immune response, e.g. *MHC* and *IFN-γ* genes. Several studies confirmed markers associated with GIN resistance close to *MHC* (Miller and Horohov, 2006, Bolormaa et al., 2010, Alba-Hurtado and Muñoz-Guzmán, 2012) and *IFN-γ* genes (Coltman et al., 2001, Crawford et al., 2006, Miller and Horohov, 2006, Bolormaa et al., 2010, Alba-Hurtado and Muñoz-Guzmán, 2012). Although, no causative mutations have been identified for published QTL studies, *IFN-γ* and *MHC* have been proposed as plausible functional and positional candidate genes (Stear et al., 2009). In contrast to the classical selection, the marker-assisted selection can utilise identified QTL to accelerate selection even in cases where the desirable alleles for the trait are found in low frequencies.

Several QTL on different regions and chromosomes (OARs) have been reported in the literature for sheep, indicating a polygenic nature for the trait (OAR1, 3, 6, 14 and 20) (Beh et al., 2002, Dominik, 2005, Crawford et al., 2006, Davies et al., 2006, Matika et al., 2011, Salle et al., 2012). In a few studies, some potential candidate genes were identified on OAR8 (Crawford et al., 2006), OAR13 (Beraldi et al., 2007), and OAR22 (Silva et al., 2012). The lack of consensus across studies may be due to parasite resistance being a genetically complex trait (Kemper et al., 2011, Riggio et al., 2013) as well as other reasons discussed in the following section.

### 2.3 Inconsistencies across studies

The lack of consistency across the results of nematode studies may be in part due to the weaknesses associated with the use of different methods of evaluation. The candidate gene approach relies on prior knowledge, however, a large majority of genes have their functions yet to be defined (Singh et al., 2014). In addition, previously identified QTL seem to disappear with new ones emerging between populations. A possible explanation for this is the differences in the analytical or experimental approaches used in different studies. Examples of these include the use of within-family microsatellite-based linkage (Beraldi et al., 2007, Gutiérrez-Gil et al., 2009, Marshall et al., 2013) *vs.* LD approaches using SNPs in genome-wide association studies (GWAS) (Riggio et al., 2013). Most of the published QTL studies were conducted using half-sib family experimental designs which uses within family linkage as opposed to a population LD. Other factors that may also contribute to these inconsistencies could be the animal population studied (i.e., different breeds, age, sex, immune and physiological status), the sample size, the nature of infection (i.e. natural infection *vs.* artificial challenge), climatic conditions (i.e. wet *vs.* dry, tropical *vs.* temperate), the production system (i.e. extensive *vs.* intensive), nematode species and the indicator traits measured.

Despite the added advantages of utilising QTL as a means of increasing genetic progress, there are still practical problems associated with the use of genetic markers as no major QTL have been identified associated with GIN resistance (i.e. GIN resistance seem to be polygenic trait, with many loci with small effect spread across the genome).

## 3 Using GWAS to identify loci underlying variation in GIN resistance

Progress in genomics along with advances in technology, statistical techniques and bioinformatics have led to the implementation of GWAS which aim at understanding the genetic basis of complex traits, such as resistance to diseases and production traits (e.g. growth, feed intake and milk yield). Previous FEC studies utilising within family linkage have been criticised for the inability to replicate results. GWAS aim at overcoming some of these limitations by searching the whole genome for genetic variants associated with quantitative traits, without prior assumptions, thus limiting bias (Hirschhorn and Daly, 2005). In cases where there is no strong evidence for a positional candidate, LD is exploited to further refine the location of the QTL to target functional mutations in causal genes (Raadsma and Fullard, 2006). The SNP arrays such as the GoatSNP50k chip with a capacity to genotype 52,295 SNPs (Tosser-Klopp et al., 2014) and OvineSNP600k chip with a capacity to genotype 603,350 SNPs (Anderson et al., 2014) are becoming important tools for GWAS. Setting up GWAS for parasite resistance requires genotyping and phenotyping large numbers of animals to obtain sufficient sample sizes (McCarthy et al., 2008). Other methods can be used to search for QTL, such as the Wright’s fixation index (F_ST_), which utilises allele frequencies between resistant *vs.* susceptible individuals and measures the degree of population differentiation. Comparisons of F_ST_ from different parts of the genome can also provide insights into the demographic history of populations and selective sweeps (Kijas et al., 2012). Few studies have been published on host resistance to nematodes in small ruminants, mostly in sheep, using SNP chips (Table 4).

### 3.1 Limitations of the GWAS methodology

In most cases, SNP chips failed to replicate results previously obtained using microsatellites. As already discussed above, the discrepancies maybe due to different factors, such as the method used (linkage analysis where markers are phased within families *vs.* LD), SNP density, lack of LD between markers and causative mutations, the breeds being analysed (which may not be well represented in the reference populations used to create the SNP chips), the polygenic nature of the traits of interest, and sample size. Large confidence intervals in the linkage analyses make it difficult to compare the results across studies (Höglund et al., 2012). Manolio et al. (2009) reported the problem of missing heritability in GWAS for complex traits. Missing heritability refers to heritability estimates of complex traits that cannot be accounted for by use of markers in GWAS, but may be attributable to non-additive genetic variances such presence of copy number variants (CNV) and epigenetics (for a detailed review on missing heritability see Vinkhuyzen et al., 2013). A *meta*-analysis conducted by Riggio et al. (2014a) highlighted how some of the challenges could be addressed. This involves the aggregation of data from several independent studies, thereby increasing power of detection of genetic variants with small effects. Work done by Kemper et al. (2012) also highlighted how some of the differences between GWAS and family-based linkage studies can be overcome, i.e. through adjusting differences in LD, and fitting all markers simultaneously instead of individually.

### 3.2 Challenges of setting up GWAS in low-input/output smallholder systems

The first hurdle in conducting GWAS in low-input/output smallholder systems, where records are scares, is obtaining accurate indicator traits. Other challenges include cases of co-infection, mixed or poorly defined breeds, and requirements for large sample sizes (Hayward, 2013). Selective genotyping and selective DNA pooling can be done to reduce number of individuals to be genotyped; however this may lead to loss of individual information (Singh et al., 2014). In low-input/output smallholder systems it may not be feasible to meet some of these requirements. In general, it is not possible to extrapolate results across distantly related populations. The genetically fragmented nature of sheep and goat populations/ecotypes makes it challenging to use the results on anything other than the population in which they are derived.

One of the key shortcomings of using the SNP technology in low-input/output systems is the cost associated with it. To mitigate this, one could exploit the advantages of imputations, in which key individuals are genotyped using higher SNP chips or sequenced to form the basis from which animals genotyped with low density SNP are imputed to the same density as the former. The power for detection of genetic associations can also be improved by performing 2-stage joint analyses which involve genotyping a proportion of the available samples in the first stage and the remaining in the second stage, with the second stage acting as replication (Skol et al., 2006). Furthermore, data sets from different studies can be combined and data imputation (after rigorous data checking) can be used as a tool to avoid bias and false-negative results (Ioannidis et al., 2009).

## 4 Application of genome-wide SNP data in parasite resistance

### 4.1 Selective sweeps/signatures

The term selective sweeps/signatures refers to advantageous alleles being fixed in a population on a particular haplotype background due to selection, leading to changes in gene frequencies of variants associated with traits (Gurgul et al., 2014). Statistical methods used for detecting selective sweeps are the F_ST_ (Weir et al., 2005), LD approach, extended haplotype homozygosity (EHH) test, integrated haplotype score (iHS), long-range haplotype (LRH) (Qanbari et al., 2011) and cross population EHH (XP-EHH) test. The XP-EHH detects selective sweeps in which the selected allele has different frequency to the other population. A study by McRae (2012) using selective sweeps on loci associated with resistance or susceptibility to GIN infection identified nine regions showing the highest signals in both Romney and Perendale lines. In another GWAS study on divergent lines selectively bred for high and low FEC, McRae et al. (2014) identified sixteen regions harbouring candidate genes associated with immunological responses to parasite infection i.e. Chitinase activity and cytokine response.

### 4.2 Copy number variation (CNV)

CNVs are defined by DNA segments which are 1 kb or larger and have variable numbers of copies to those in the reference genome (Iafrate et al., 2004). These variants exhibit similar demographic patterns to SNPs. CNV analysis on genome-wide SNP data can lead to identification of chromosomal regions containing structural variations affecting complex traits (Zarlenga and Gasbarre, 2009). A GWAS between CNVs and resistance to GINs in Angus cattle resulted in haplotype blocks containing immune-related genes being detected (Xu et al., 2014). According to these authors, when the CNV co-segregates with linked SNPs and associated genes, it contributed to the detected variations in gene expression and thus difference in host parasite resistance. Studies in sheep performed to investigate differentially expressed genes (DEGs) have identified various DEGs related to parasite resistance (Diez-Tascon et al., 2005, Keane et al., 2006, Ingham et al., 2016). A study conducted by Liu et al. (2011) in cattle identified 20 CNVs, 85% of which were associated with parasite resistance. Another large scale analysis of CNVs using SNP genotyping data by Hou et al. (2012) detected 297 CNV regions which were validated by qPCR and overlapped with 437 Ensembl genes associated to GIN infection. Current high-throughput genome scan technologies such as next-generation sequencing (NGS) or SNP genotyping microarrays enables CNV identification at a genome-wide scale (Gheyas and Burt, 2013). The NGS has a potential of reducing ascertainment bias. Despite some of the highlighted potentials, these technologies have not been applied widely to small ruminants.

### 4.3 Genomic selection

As discussed above, classical genetic improvement programmes have relied on the use of phenotypes and pedigree information to generate estimated breeding values (EBV). The increasing use of SNP markers in studying complex traits also avails the potential to calculate genomic estimated breeding values (GEBVs) for traits such as parasite resistance when adequate genotypes and phenotypes are available. Understanding genetic architecture underlying resistance will enable the prediction of genetic risk or selective breeding (Spencer et al., 2009, Hayes et al., 2010). The genomic selection approach was first proposed by Meuwissen et al. (2001) in an attempt to use all SNPs in predictions and has since become a powerful tool especially for genomic predictions in polygenic traits. Furthermore, the accuracy of estimated SNP effects is influenced by the size of the reference population and genetic variance is explained by markers influenced by the effective population size (Ne) and the density at which the SNP chip covers the genome.

The accuracy of GEBV has been evaluated in experiments involving different livestock species, including sheep (Daetwyler et al., 2012, Riggio et al., 2014b). For reliable genomic prediction, the population under evaluation should have a close relationship with the reference population (Habier et al., 2010). To date, limited studies have been reported on the use of high density genomic information to select for nematode resistance in small ruminants. This may be due to low animal value, and high cost of genotyping. According to Kemper et al. (2011), genomic prediction of nematode resistance suggests only moderate accuracy with currently available SNP arrays; however, the potential of genomic selection warrants that the concept be further investigated. Riggio et al. (2014b) have reported moderate accuracies in a within breed approach; however, they also reported that across breed accuracies were low or close to zero. Within breed genomic selection provides the benefits such as improved genetic progress and reduced generation interval. Genomic selection is now well established in the dairy cattle sector (for milk production) with examples in New Zealand and Ireland where GEBVs are now being routinely used by farmers (Spelman et al., 2013). The genomic selection programmes for sheep are starting to be rolled out to farmers also in Australia. This may require cheap genotyping (low density SNP chip) of large numbers of animals combined with imputation from high density information in targeted animals in order to facilitate predictions across breeds (Van der Werf, 2009). This could be a potential tool for low-input/output farming systems, in which well phenotyped and genotyped animals from the same “breed” could be used as the training set to predict village animals genotyped using a lower coverage of 5k SNPs or less. Imputation from the 5k to 50k or higher SNP coverage can then be done to allow better prediction. The other option would be to create low density “custom” SNP chip which then incorporates the main GWAS hits from genome-wide association *meta*-analyses studies. Such an approach was successful in human data albeit the “hits” were generated from high powered studies (Spiliopoulou et al., 2015). These approaches will have the potential to reduce costs; however no low density arrays are commercially available. For now, the challenges of setting up such breeding schemes are great and genomic selection at least with current technologies, is likely to be expensive and logistically difficult to implement in tropical sheep and goats. Despite all these limitations, in systems where records are scarce, genomic selection is the only tool that still offers real potential in improving breeding. In these scenarios, a few farmers can be incentivised to collect data which then can be used to predict genetic merit from non-recorded communal flocks.

### 4.4 Integrated control, eradication to manipulation of host-parasite equilibrium

Anthelmintic resistance in nematode populations may have resulted in part from the recurrent use and over reliance on drugs. As a result of this, concerns have been raised as to whether host genetic resistance would similarly breakdown over a period of time, with nematodes evolving to adapt to the resistant hosts (McManus et al., 2014). According to Bishop (2012), the polygenic nature of host-parasite resistance suggests that worm evolution should be slower than that of anthelmintic resistance, as worms would have to evolve against many more targets. In addition to that, there is no published evidence for apparently resistant breeds losing their relative advantage compared with those that are more susceptible.

The strategy of nematode control has evolved to a more logical manipulation of host-parasite equilibrium in grazing systems by implementation of various actions, which include genetic resistance of small ruminants. According to Mandonnet et al. (2014), different strategies can be implemented for nematode control especially in the tropics; these include short-term strategies like reducing host contact with infective larvae through grazing management. It also involves other strategies such as extending the efficiency of current AH molecules through targeted selective treatments (TSTs) which rely on the assumption that some animals are more infected than others. In addition to that, it also relies on a longer-term strategy which involves enhancing the ability of the host to tolerate the negative effects of worm through genetic selection.

Use of markers in genomic selection dispenses with the need to record pedigrees since these can be reconstructed from the markers. However, accurate phenotypes for the reference populations will still need to be collected. These could be through creating some “phenotype farms” where farmers are incentivised to collect the phenotypes. Some possibilities would be to use existing Research institute facilities or form breeding schemes (in low-input/output smallholder farming systems) through centralised nucleus flocks and village or community-based flocks. By using these strategies, the problems associated with cost of using genomic tools may be mitigated. Village flocks can then be improved for parasite resistance using the genetic merits of the animals in the nucleus flock. Selection for resistant hosts can thus be considered as a sustainable control strategy because it leads to reduced pasture contamination and increased overall flock productivity. However, whatever method will be implemented, success will be most likely to be achieved if they are used to complement other control strategies.

## 5 Conclusions

Different control strategies can be put in place and these include improved nutrition, reducing host contact with infective stages, use of vaccination, extending efficiency of anthelmintic through target selective treatment and in the long term enhancing the ability of the host to tolerate negative effects of the worm. Given the reviewed candidate gene, QTL mapping and GWAS studies, the genetic architecture of GIN is a trait influenced by many loci with small effects. The overall lack of consensus in different studies can be explained by the diversity in studies involving different breeds, parasites species and experimental procedures.

The use of sustainable genetic tools is not the ultimate solution but its use in combination with other integrated control methods could yield positive results. Conventional breeding systems involves phenotyping traits of importance and based on information available on the pedigree, EBVs are computed and used as a basis of selection. The use of genomic tools has the potential to be explored in low-input/output farming systems, where no records are kept. The identification of SNPs associated with GIN resistance can be used to develop customised chips for the low-input/output farming systems. In the long-run it is possible to consider the use of genomic tools as an alternative means of parasite control.

## Figures and Tables

**Table 1:** Cases of anthelmintic resistance in sheep and goats.

Species	Country	Anthelmintic^a^ (Class)	Nematode genera	Reference(s)
Goats	Ethiopia	Albendazole, Tetramisole, Ivermectin (BZ, IMID, AVM)	*H. contortus, Trichostrongylus, Teladorsagia spp*	Sissay et al., (2006), Kumsa and Abebe (2009)
	Uganda	Albendazole, Levamisole, Ivermectin (BZ, IMID, AVM)	*H. contortus, Cooperia spp. Oesophagostomum spp*	Byaruhanga and Okwee-Acai, 2013
	Nigeria		*H. contortus*	Chiejina et al. (2010)
	Pakistan	Oxfendazole, Levamisole (BZ, IMID)	*H. contortus, T. colubriformis*	Saeed et al. (2010)

Sheep	Zimbabwe	Fenbendazole, Albendazole, Oxfendazole, Levamisole (BZ, IMID)	*H. contortus, Cooperia* spp.	Mukaratirwa et al. (1997), Matika et al. (2003)
	Zimbabwe	Fenbendazole, Levamisole, Rafoxanide (BZ, IMID, SCL)	*H. contortus*	Boersema and Pandey (1997)
	Zambia	Ivermectin, Albendazole (AVM, BZ)	*H. contortus*	Gabriel et al. (2001)
	Germany	Levamisole, Ivermectin (IMID, AVM)	*Trichostrongylus spp*	Voigt et al. (2012)
	Brazil	Ivermectin (AVM)	*H. contortus,*	Fortes et al. (2013)
	Northern Ireland	Benzimidazole, Moxidectin, AvermectinLevamisole (BZ, MLB, AVM, IMID)	*Trichostrongylus Teladorsagia, Cooperia* spp.	McMahon et al. (2013)

Sheep/goats	South Africa	Albendazole, Closantel, Ivermectin, Levamisole (BZ, SCL, AVM, IMID)	*H. contortus, Trichostrongylus, Oesophagostomum spp*	Bakunzi et al. (2013), Tsotetsi et al. (2013)
	Kenya	Ivermectin,Fenbendazole (AVM, BZ)	*H.contortus, Trichostrongylus,Oesophagostomum* spp.	Mwamachi et al. (1995)
	Switzerland	Avermectin (AVM)	*Haemonchus contortus, Trichostrongylus spp*	Artho et al. (2007)
	Norway	Albendazole (BZ)	*Teladorsagia, Trichostrongylus spp*	Domke et al. (2012)
	India	Fenbendazole, Benzimidazole (BZ)	*H. contortus, Trichostrongylus spp*	Rialch et al. (2013)
	India	Thiabendazole, Tetramisole (BZ, IMID)	*H. contortus*	Swarnkar and Singh (2011)
	Philippines	Benzimidazoles (BZ)	*H. contortus*	Ancheta et al. (2004)

a Benzimidazoles—BZ; Macrocyclic lactones- ML (Avermectins-AVM or Milbemycin−MLB; Nicotinic agonists (Imidothiazoles-IMID or Tetrahydropyrimidines-TETR); Aminoacetonitriles derivatives-AAD; Salicylanilides-SCL.

**Table 2:** Small ruminant breeds with reported resistance traits against gastrointestinal parasites.

Species	Resistant Breed	Susceptible breed	Infection^a^	Parasite(s)^b^	References
Goats	Sabi	Dorper	N	Hc	Matika et al. (2003)
	Small East African (SEA)	Galla	N	Hc	Baker et al., 1994, Baker et al., 1998
	Jamunapari	Barbari	N	Hc, *Strongyloides Oesophagostomum spp*	Rout et al. (2011)
	Creole	–	N	Hc, Tc	Mandonnet et al. (2001)
	Creole	–	A	Hc	Bambou et al. (2009)
	Creole	–	N	Hc	de la Chevrotiere et al. (2012a)
	West African	–	N	Mixed	Behnke et al. (2011)

Sheep	Gulf Coast Native	–	N	Hc	Peña et al. (2004)
	F_1_ and F_2_ SuffolkX Gulf Coast Native	–	N	Hc	Li et al. (2001), Miller et al. (2006)
	INRA 401	–	A	Hc, Tc	Gruner et al. (2004)
	Merino	–	A	Hc, Tc	Andronicos et al. (2010)
	Gulf Coast Native	Suffolk	N	Hc, Tc	Miller et al. (1998), Shakya et al. (2009)
	Red Masaai	Blackheaded Somali, Dorper, Romney Marsh	A/N	Hc	Mugambi et al. (1997)
	Barbados black belly	INRA401	A	Trichostrongyles	Gruner et al. (2003)
	Santa Ines	Ile de France, Suffolk	N	Hc, *Oesophagostomum columbianum*	Amarante et al. (2004)
	Texel	Suffolk	N	T*richostrongyle; Teladorsagia, Nematodirus*	Sayers et al. (2005); Good et al. (2006)
	Florida native, Florida native X Rambouillet	Rambouillet	N	Hc	Amarante et al. (1999)
	Dorper X Katahdin	Hampshire	A/N	Mixed	Burke and Miller (2002)
	Lohi	Thalli, Kachhi	A/N	Hc	Saddiqi et al. (2010)
	Caribbean Hair, Katahdin	Crossbred-Dorper	A	Hc	Vanimisetti et al. (2004)

(−) Indicates trials which only involved one breed, within-breed differences.

^a^N—natural infection; A—artificial challenge.

^b^Hc-*Haemonchus contortus*; Tc- *Trichostrongylus colubriformis.*

**Table 3:** Feacal egg count heritability estimates (h^2^) in small ruminants.

Species	Breed(s)	h^2^	Age (mo)	Country	References
Goats	Galla and SEA	0.13	4.5–8	Kenya	Baker et al. (1994)
	Cross-bred Cashmere	0.2–0.3	12–18	Scotland	Vagenas et al. (2002)
	Creole	0.14–0.33	4–10	French west indies	Mandonnet et al. (2001)
	Creole	0.10	>11	French west indies	Mandonnet et al. (2006)

Sheep	Dorper vs Red Masaai	0.18 vs. 0.35	8	Kenya	Baker (1998)
	Menz and Horro	0.01–0.15	1–12	Ethiopia	Rege et al. (2002)
	Rhon and German Merino	0–0.35	3–5	Germany	Gauly et al. (2002)
	Merino	0.2–0.65	4–13	Australia	Pollot et al. (2004)
	Dorset-Rambouillet-Finn(Lambs–ewes)	0.15–0.39	4 (1–10years)	Australia	Vanimisetti et al. (2004)
	Soay	>0.10–0.26		Scotland	Beraldi et al. (2007)
	Santa Ines lambs	0.01–0.52	–	Brazil	Lôbo et al. (2009)
	Scottish Blackface	0.14	6–7	Scotland	Stear et al. (2009)

**Table 4:** Published QTL studies on host resistance to nematodes in small ruminants.

Species	Markers^a^	Breed	Chromosome	References
Goats	M	Australian Angora and Cashmere	23	Bolormaa et al. (2010)
	M	Creole	22, 26	de la Chevrotiere et al. (2012b)
Sheep	M	Romney- Coopworth	8, 23	Crawford et al. (2006)
	M	Scottish Blackface	2, 3, 14 and 20	Davies et al. (2006)
	M	Soay	1*, 6*, 12*	Beraldi et al. (2007)
	M	Scottish Blackface	3, 20	Stear et al. (2009)
	M	Spanish Churra	1, 6, 10, 14	Gutiérrez-Gil et al. (2009)
	SNP	Merino		Marshall et al. (2009)
	M	Romney-Merino Backcross	3*, 21, 22*	Dominik et al. (2010)
	M	Suffolk and Texel	3, 14	Matika et al. (2011)
	M, SNP	Romane-Martinik Blackbelly Backcross	5, 12, 13, 21	Salle et al. (2012)
	M	Red Masaai, Dorper	2, 26	Marshall et al. (2013)
	SNP	Soay	1, 9*	Brown et al. (2013)
	SNP	Scottish Blackface	6, 14	Riggio et al. (2013)
	SNP	Scottish Blackface, Sarda-Lacaune Backross, Martinik Blackbelly-Romane Backcross	4*, 6, 14, 19*, 20*	Riggio et al. (2014a)
	SNP	Red Maasai-Dorper Backcross	6, 7	Benavides et al. (2015)

*Suggestive associations.

a M−Microsatellites; SNP−OvineSNP50 chip.
